# MeCP2 and Chromatin Compartmentalization

**DOI:** 10.3390/cells9040878

**Published:** 2020-04-03

**Authors:** Annika Schmidt, Hui Zhang, M. Cristina Cardoso

**Affiliations:** Department of Biology, Technical University of Darmstadt, 64287 Darmstadt, Germany; annika.schmidt.mail@gmail.com (A.S.); zhanghui20139@gmail.com (H.Z.)

**Keywords:** DNA modifications, DNA methylation readers, higher order chromatin structure, heterochromatin, MeCP2, Rett syndrome

## Abstract

Methyl-CpG binding protein 2 (MeCP2) is a multifunctional epigenetic reader playing a role in transcriptional regulation and chromatin structure, which was linked to Rett syndrome in humans. Here, we focus on its isoforms and functional domains, interactions, modifications and mutations found in Rett patients. Finally, we address how these properties regulate and mediate the ability of MeCP2 to orchestrate chromatin compartmentalization and higher order genome architecture.

## 1. Introduction

In humans, the two meter long genomic DNA is hierarchically folded to fit inside the membrane-bound micrometer-scale cell nucleus. Individual chromosomes occupy distinct subnuclear territories. The chromosome territories have been proposed to be further subdivided into two mutually excluded compartments called ‘A’ (active) and ‘B’ (inactive) with distinct accessibilities. Each compartment was reported to consist of multiple topologically associating domains (TADs) (reviewed in [1]). Within TADs, DNA/chromatin looping was predicted to promote higher DNA interaction frequencies among DNA sites located far apart within the linear DNA molecule (reviewed in [1]).

Epigenetic chromatin modifications, including DNA and histone modifications, were shown to control genome accessibility [2] and, thus, the spatial-temporal gene expression without changing the nucleotide sequence. DNA methylation, established by DNA methyltransferases, blocks the access of multiple factors to DNA, thus creating repressive regions. This DNA modification is read by methyl-CpG binding domain (MBD) protein family, which in addition recruit specific chromatin modifiers (reviewed in [3]). Methyl-CpG binding protein 2 (MeCP2) was the first member of the MBD family to be identified [4] and the most extensively studied one. Hereafter, we will focus on MeCP2 isoforms, domains, interactions, modifications and mutations before moving to its role in higher order chromatin organization.

## 2. MeCP2 Interactions, Modifications and Mutations

### 2.1. MeCP2 Isoforms and Domains

The *MeCP2* gene is highly conserved in Euteleostomi (bony vertebrates) and in humans is located on the X chromosome. Mutations in the *MeCP2* gene were linked to the human neurological disorder Rett syndrome (RTT) [5]. The MeCP2 protein has two isoforms (MeCP2 e1 (exon 1) and MeCP2 e2 (exon 2)) with different amino termini due to alternative splicing and different translational start sites. The two isoforms of MeCP2 are abundantly expressed in the central nervous system, but with different expression levels and distributions in developing and post-natal mouse brains. MeCP2 e1 is the predominant isoform in brain and has an earlier expression onset than MeCP2 e2 [6]. The two isoforms are commonly considered as functionally equivalent, yet recent evidence shows that MeCP2 e1 plays a role in neuronal maturation [7] and is more relevant for RTT [8,9,10]. In view of the fact that MeCP2 e2 isoform was the first to be known and a much larger body of literature pertains to this isoform, we will, throughout, use amino acid coordinates from MeCP2 e2 isoform.

**Figure 1:**
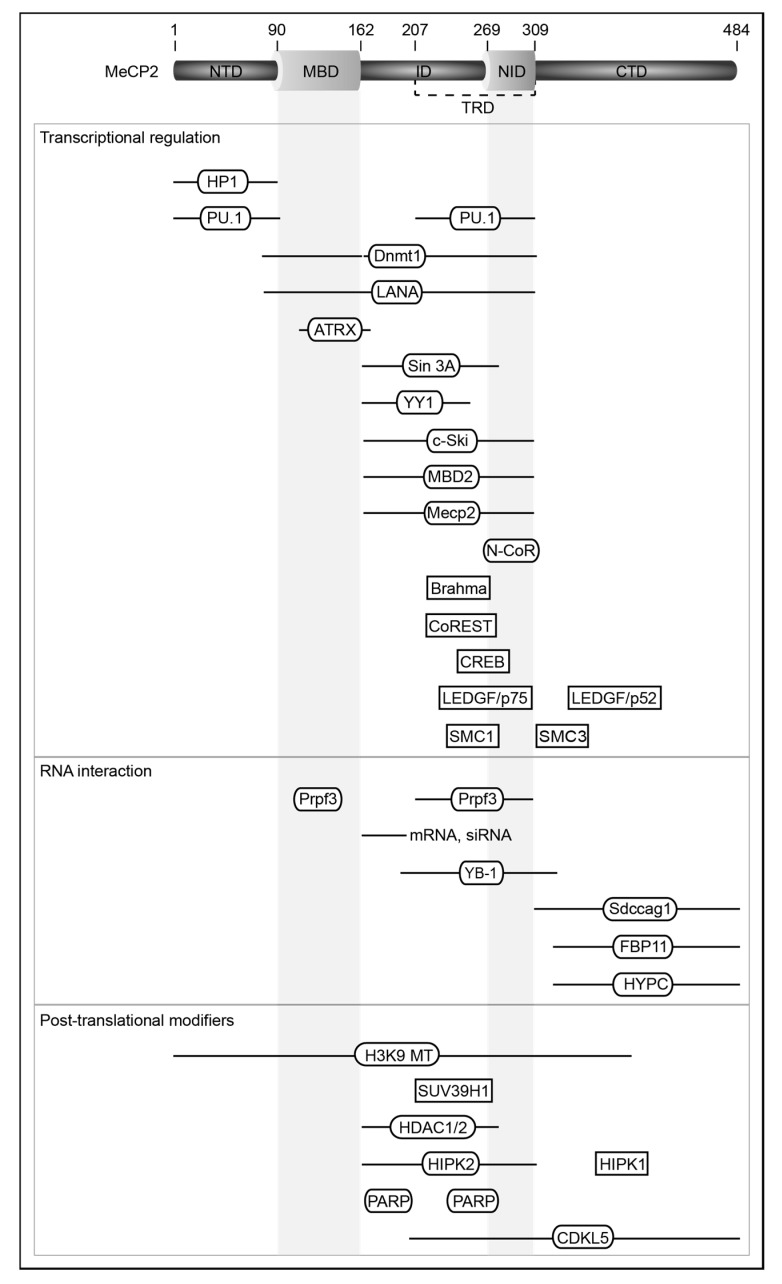
Overview of MeCP2 interaction partners. MeCP2 interaction partners, group by main function and ordered by where they interact within MeCP2, if known. References are given in Table 1. Rectangles indicate proteins with no mapped interaction region within MeCP2. NTD: N-terminal domain; MBD: methyl binding domain; ID: intervening domain; NID: N-CoR interacting domain; CTD: C-terminal domain; TRD: transcriptional repression domain. Amino acid labeling according to mouse MeCP2 isoform e2. Protein domain structure generated using DOG 1.0 software [61].

Both variants include two functionally characterized domains: the methyl-CpG binding domain (MBD) and the transcriptional repression domain (TRD). The MBD specifically recognizes and binds 5-methylcytosine (5mC), while the TRD was found to bind multiple transcriptional repressors, thus silencing gene expression [11,12,13,14,15,16]. However, the TRD was also shown to bind to multiple transcriptional activators and activate gene expression [17,18,19]. More recently, the TRD has been narrowed down to the N-CoR/SMRT interacting domain (NID) [20]. A summary of the best characterized domains of MeCP2 is shown in Figure 1. The DNA binding properties of the different domains and the mechanism of DNA binding will be addressed in the next section.

### 2.2. MeCP2 DNA Dinding

Early studies on MeCP2 characterized it as a protein being capable to bind to a single, symmetrically methylated CpG pair via the MBD domain spanning amino acids 89 – 162 and thereby overlapping approximately twelve base pairs of DNA [4,21,22]. Later studies indicated that the N-terminal domain (NTD) enhanced DNA binding affinity via the MBD [23], while the intervening domain (ID), TRD and C-terminal domain (CTD) alpha showed methylation-independent DNA binding capabilities and CTD beta was proposed to bind to chromatin, but not to naked DNA [23,24]. Furthermore, three AT-hook-like domains were identified within the ID, TRD and CTD alpha domains (AT-hook 1, aa 184–195; AT-hook 2, aa 264–273; AT-hook 3, aa 341–364). The AT-hook motif is a short motif binding to the minor groove of AT-rich DNA via the core consensus amino acid sequence RGRP [25]. These methylation-independent DNA binding capabilities allow MeCP2 to bind to different sites on the DNA at the same time, thus, possibly contributing to genome-wide chromatin organization. With the exception of the MBD, MeCP2 was shown to be mostly an intrinsically disordered protein. Upon binding to DNA, though, increased secondary structure in ID and TRD were observed [23]. The MBD is the only domain showing structurally conserved motifs, as it contains four beta-sheets and one alpha-helix building up a wedge shape with a beta-sheet face presenting positively charged amino acids for interaction with the DNA as determined by nuclear magnetic resonance analysis [26]. Accordingly, this domain showed only minor conformational changes as a result of DNA binding [23,26]. The subsequent crystal structure of the MBD bound to the *Bdnf* gene promotor revealed that MBD mCpG interaction might involve five water molecules, leaving only three amino acids with direct contact to the DNA: D121, R111 and R133 [27]. In line with this study, these amino acids were found mutated in RTT and with significantly reduced MeCP2 DNA binding [27,28,29].

Dynamic structural analysis of MeCP2 using H/DX-MS, led to the proposal that the intrinsically disordered MeCP2 samples multiple conformational states, also during non-specific interaction with the DNA [30].

Using genome wide chromatin immunoprecipitation-sequencing (ChIP-seq) analysis, MeCP2 was found to bind globally across the genome tracking mCpG density [31]. Furthermore, in purified nuclei from mouse brain MeCP2 was shown to be expressed at near histone octamer levels [31]. These findings suggest that MeCP2 binds globally across the genome reducing transcriptional noise.

Nevertheless, MeCP2 was also described to bind to actively transcribed unmethylated DNA in vivo [17,32] with only a minor portion of MeCP2-bound promoters being highly methylated [32]. A possible explanation would be that MeCP2 folds upon binding to DNA and scans the DNA for suitable binding sites making use for this of its non-specific DNA binding sites [23,33]. Thus, it would only bind non-specifically to active genes to scan the DNA for mCpG binding sites.

Recently, MeCP2 was reported to bind not only mCpG but also mCpApC [34]. The patterns of mCpApC differ between neuronal cell types and may, thus, contribute to cell type specific effects of MeCP2 [35,36].

In addition to binding DNA and methylated cytosines, MeCP2 was proposed to bind to 5-hydroxymethylcytosine (5hmC) in mouse brain [37] and embryonic stem cells [38]. 5hmC is an oxidation product of 5mC and can be further oxidized to 5-formylcytosine (5fC) and 5-carboxylcytosine (5caC) by TET (ten-eleven-translocation) proteins, which might enable active DNA demethylation by different pathways (reviewed in [39]). In addition, 5hmC levels were reported to be differentially distributed between different tissues, much lower than 5mC levels and associated to actively expressed and developmentally regulated genes [40]. Nevertheless, these findings are highly debated, as the results are tissue and cell type dependent [37,38], the recognition mechanism of 5hmC by MeCP2 is unclear and other studies hint to a binding affinity similar to binding unmethylated DNA [41,42,43].

A more indirect way of MeCP2 to repress transcription by DNA binding is the protection of MeCP2 bound 5mC against oxidation to 5hmC by TET enzymes by restricting their access to the methylated cytosine [44]. This was proposed to contribute to restricting transcriptional noise [31] and, in particular, repressing tandem repeat DNA expression [44] and L1 retrotransposition [45,46,47]. TET-mediated L1 activation was shown to be prevented by binding of MeCP2 to 5mC [47].

Summarizing, methylation-specific and unspecific MeCP2 DNA binding are both essential for its function in transcriptional repression and chromatin organization, and its multifunctional domain structure allows the protein to simultaneously bind to DNA and interact with other proteins, which will be described next.

### 2.3. MeCP2 Protein–Protein Interactions

Interactions of MeCP2 with several proteins mediate and regulate its multiple functions in transcriptional regulation, chromatin organization and RNA splicing. An overview of interacting proteins, the interacting MeCP2 regions and the function of these interactions is presented in Figure 1 and Table 1.

One major mechanism by which MeCP2 represses transcription is by recruiting corepressor complexes to methylated DNA. One such complex contains mSin3A and histone deacetylases (HDACs), suggesting that transcriptional repression may in part rely on histone deacetylation [11,12], e.g., by removing active chromatin marks. mSin3A was shown to be the direct MeCP2 binding partner, whereas HDACs showed a weaker binding affinity to MeCP2 and, thus, might bind via mSin3A [11]. Another corepressor complex reported to interact with MeCP2 is the NCoR/SMRT interacting with a small region within the TRD domain, which was thus called NID. The data suggested that MeCP2 recruited NCoR/SMRT to methylated DNA and that this MeCP2 bridge function is disturbed in RTT [20]. Interestingly, binding of Sin3A was not disrupted by NID mutations [20].

In addition to transcriptional repression, MeCP2 might also work as an activator, as it was found associated with the transcriptional activator CREB1 (cyclic AMP-responsive element-binding protein 1) at the promoter of an activated gene [17]. In gene expression analysis from mouse hypothalami, the gain of MeCP2 was shown to result in more transcriptional activation than repression, whereas MeCP2 loss lead to reverse effects [17]. These results are in line with a previous study, where only a minor portion of MeCP2 was found bound to methylated CpGs, but 63% of MeCP2 were bound to actively expressed promoters [32]. In other studies though, MeCP2 was found to track methylated CpGs genome wide [31], as described above.

As transcriptional activity is influenced by chromatin organization, these MeCP2 functions can hardly be separated. By interacting with histone methyltransferase acting on histone H3 lysine 9, MeCP2 was reported to target histone methylation to methylated regions on the DNA [48]. As mentioned above, MeCP2 transcriptional repression involves recruitment of histone deacetylases and deacetylation of histones is likely followed by histone methylation [48], thus switching chromatin from an active to a repressive state. Histone methylation may result in recruitment of other proteins like heterochromatin protein 1 (HP1), thus reinforcing the repressed chromatin state [49,50]. MeCP2 and HP1 were shown to interact [51] and both were reported to associate with SUV39H1 (suppressor of variegation 3-9 homolog 1) histone methyltransferase [13,52], which methylates histone H3 lysine 9. In addition, MeCP2 might be involved in regulation of maintenance DNA methylation by DNMT1 (DNA methyltransferase 1), as the interaction of both proteins was also described [53]. DNMT1 interacts with HDAC1 and 2 [54,55], and was shown to replace the mSin3A-HDAC complex upon MeCP2 binding [53].

Another mechanism by which MeCP2 modulates chromatin architecture could be oligomerization. In that regard, MeCP2 was shown to associate with itself and the methyl CpG binding domain protein 2 (MBD2) [56]. Furthermore, MeCP2 associates with the chromatin remodeling protein ATRX (alpha-thalassemia/cognitive disability syndrome X-linked). Analysis of MeCP2 null mouse brains showed delocalization of ATRX from heterochromatic foci, suggesting a MeCP2-dependent ATRX targeting to heterochromatic regions in mature neurons [57]. As the MeCP2 mediated ATRX targeting to heterochromatin took place only in mature neurons where MeCP2 is very abundant [57], this underscores the relevance of MeCP2 level for its function.

MeCP2 might also play a functional role in RNA splicing, as it binds to WW domains of the splicing factors FBP (formin-binding protein) 11 and HYPC (Huntington yeast partner C) via a proline rich domain in the MeCP2 C-terminus [58,59]. Genotype-phenotype studies on RTT frameshift mutations support the hypothesis that disruption of the proline-rich region in the MeCP2 C-terminus, thus abolishing its binding to FBP11 and HYPC, contributes to Rett phenotype [59]. In addition, association of MeCP2 with the Y box-binding protein 1 (YB-1), a conserved DNA and RNA binding protein [60], promotes exon inclusion in YB-1 responsive CD44-splicing reporter assays [60]. This leads to the proposal that misregulation of transcription as well as splicing might contribute to RTT [60].

Although several MeCP2 interaction partners were identified so far, the whole network of protein-protein interactions, their interplay and the entire composition of MeCP2 transcription silencing compartments require further investigation. Importantly, MeCP2 DNA binding and protein-protein interactions need to be studied in the context of post-translational modifications as these can abolish or enhance DNA and protein binding, thus, ultimately influencing chromatin organization.

### 2.4. MeCP2 Post-Translational Modifications

Recently, several MeCP2 post-translational modifications (PTMs) were reported, mostly in large scale proteomic studies focusing on mapping one specific PTM in the whole proteome. In Table 2, experimentally determined MeCP2 modifications are summarized, together with the species in which they were identified, the methods used for identification along with references. A more detailed list can be found on PhosphoSitePlus.org [73], including additional sites only available as curated datasets.

Most of the modifications were identified in large scale studies and not further validated by any other assay. Furthermore, in most cases no additional information is available regarding their influence on MeCP2 function (e.g., [74,75,76,77,87,88,92]). Many of these PTMs were mapped using a single cell line (e.g., [74,77,88]), and their existence in vivo has not been demonstrated. For these reasons, we will focus here on the more detailed studies providing validation and functional relevance of MeCP2 PTMs, in particular, within the context of chromatin.

The first phosphorylation (phos) site identified on MeCP2 was mapped to the CTD on serine 421. S421phos was found as an upshifted band on Western blot analysis upon membrane depolarization [83,120,121] and occurring exclusively in brain, although MeCP2 was detected in many other tissues [122]. S421A/S424A double mutant mice showed better performance in hippocampal memory tests, enhanced longterm potentiation [114] and increased locomotor activity [84]. Analysis of Mecp2 S421A mice revealed an increased dendritic complexity, and defects in the response to novel experiences [123]. As global S421phos was observed upon membrane depolarization, this modification might not regulate expression of specific genes, but rather be involved in modulating global response to membrane depolarization [123].

Together with S421phos, S80phos within the NTD is one of the most studied MeCP2 phosphorylation sites with functional characterization. In contrast to S421 phosphorylation, serine 80 was reported to be dephosphorylated upon membrane depolarization and S80A mutant mice show decreased locomotor activity [84]. The modification is highly enriched in the brain and ubiquitously distributed similar to total MeCP2 [84]. S80A mutation decreased MeCP2 chromatin binding affinity, although the MeCP2 S80A protein levels and subcellular distribution did not differ relative to the wildtype MeCP2. Thus, it was suggested that the phosphorylation possibly fine-tunes chromatin association [84]. The homeodomain-interacting protein kinases 1 (HIPK1) and 2 (HIPK2) were proposed to be responsible for MeCP2 phosphorylation at serine 80 [68,69].

Another MeCP2 phosphorylation site influencing chromatin binding affinity was identified on tyrosine 120 within the MBD domain of MeCP2. This tyrosine residue is substituted in a RTT patient by aspartic acid [124], which could mimic the phosphorylated state. MeCP2 Y120D mutation was found to cause a decrease in binding affinity of MeCP2 to heterochromatin [28]. This could be explained at the structural level by computational modeling indicating that MeCP2 Y120D drastically reduces MeCP2 affinity for DNA as compared to wildtype MeCP2 [91].

A conserved serine (S164) located at the beginning of ID just after the MBD, was shown to be abundantly phosphorylated in the brain in a developmentally regulated manner [97]. While the phospho-mimicking version S164D showed minor binding to chromatin in live-cell kinetic studies, the phospho-defective mutation S164A had the opposite effect [97]. These results could be explained by in silico modeling of the 3D structure of this phosphorylation site, revealing the addition of negative charge to the protein surface as a consequence of S164 phosphorylation, hence, decreasing DNA binding. Immunofluorescence analysis of wildtype neurons versus MeCP2 S164 mutants revealed that temporal regulation of S164 phosphorylation is required for proper nuclear size and neuronal dendritic branching [97].

In addition to phosphorylation, poly(ADP-ribosyl)ation (PAR) of MeCP2 at the ID and TRD domains was reported to occur in vivo in the mouse brain and to influence heterochromatin structure. The addition of this anionic modification within the two highly cationic MeCP2 protein domains responsible to bind DNA was proposed to lead to a general decrease in DNA binding affinity [70]. Concomitantly, poly(ADP-ribosyl)ation of MeCP2 was shown to reduce binding and clustering of pericentric heterochromatin in cell-based assays, suggesting a role of this PTM in MeCP2 chromatin architecture regulation [70].

Altogether, MeCP2 modifications have been shown to regulate its ability to bind and organize DNA/chromatin, as they change the molecular properties of the respective amino acids, which can be critical depending on the position of the residue within the MeCP2 domains. Yet, as mentioned above, most of the modifications identified in MeCP2 have not been functionally characterized and their role in RTT is unclear. The next section will address the consequences of MeCP2 mutations occurring in the context of RTT.

### 2.5. MeCP2 RTT Mutations

**Figure 2:**
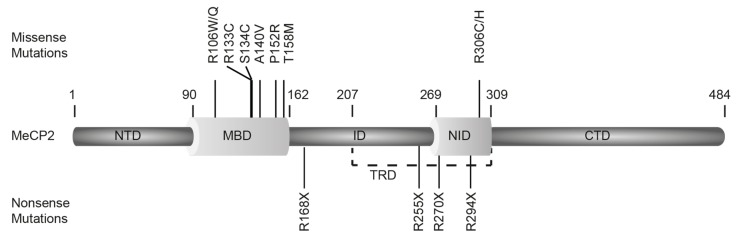
Diagram showing the high frequency mutation spectrum in Rett syndrome patients. A compendium of RTT mutations can be found in the online RettBASE. Missense mutations are shown above and nonsense mutations below the scheme showing the structure of MeCP2 (MeCP2 domains as in Figure 1). X means point mutation to stop codon, thus generating a truncated protein. Amino acids and substitutions are given according to the single-letter nomenclature. Mutation numbering according to human MeCP2 isoform starting in exon 2.

MeCP2 was shown to be associated with the neurological disorder Rett syndrome (RTT), as mutations in this gene were found in about 80% of RTT patients [5]. RTT affects mostly young girls and is characterized by normal development until 7-18 months of age, followed by a developmental stagnation and decline of higher brain functions [125]. Mutations causing RTT and related neurological disorders have been identified along the entire MeCP2 locus, but effects vary depending on the mutation type and location. Missense and nonsense mutations are the most commonly found and relatively well studied. A collection of all RTT related mutations can be found in the online RettBASE: RettSyndrome.org (http://mecp2.chw.edu.au/cgi-bin/mecp2/search/printGraph.cgi). Figure 2 graphically summarizes the high frequency mutations causing RTT (Figure 2) and Table 3 describes their phenotypes.

In the following, we will concentrate on RTT mutations impacting MeCP2 DNA binding and chromatin organization function.

MeCP2 RTT related missense mutations are largely found in the MBD, and a large proportion of these mutations reduce the 5mC binding affinity and, consequently, lead to impaired heterochromatin organization and function in cells [28].

MeCP2 R133 and R111 residues located within the MDB directly contact 5mC, and mutations at either site decrease MeCP2 localization at heterochromatin in vivo albeit to different extent. MeCP2 R111G is a rare RTT mutation found only in one patient, which abolishes MeCP2 localization to heterochromatin [28]. MeCP2 R133 mutation influences the pericentric heterochromatin localization depending on the amino acid substitution. MeCP2 R133C and R133L decrease the enrichment at heterochromatin, whereas R133H promotes it [28,29]. Furthermore, artificially targeting MeCP2 R111G and R133L mutants to pericentric heterochromatin rescued their ability to cluster heterochromatin [29].

T158 is the most frequently found MeCP2 MBD mutation site in RTT patients and two substitutions have been reported, T158M (frequency 419) and T158A (frequency 2). Neurons expressing MeCP2 T158M showed reduced neurite outgrowth and dendritic complexity by down regulating the expression and phosphorylation of transcriptional activator CREB1 [135,136]. Both MeCP2 T158M and T158A proteins show decreased stability, methyl-DNA binding ability and heterochromatin clustering function [28,128,139,145].

TRD is a second mutational hotspot domain in MeCP2. Considering its function in direct interaction with multiple transcriptional repressor complexes (see Figure 1 and Table 1), mutations within this region are considered to influence the recently proposed MeCP2 ‘bridge’ function between repressors and chromatin [20,146].

R306C is the most frequent missense mutation found within the MeCP2 TRD. Mutant mice expressing MeCP2 R306C showed typical RTT phenotype: hind limb clasping, impaired mobility and motor coordination, reduced brain weight and size [147]. This mutation did not influence the MeCP2 methyl-DNA binding ability in vitro [128], but showed decreased MeCP2 DNA occupancy in vivo [147], and lack of interaction with NCoR/SMRT [85]. R306C also abolished (neuronal activity-dependent) phosphorylation at the nearby T308 residue. The effect of losing T308 phosphorylation was tested by creating a MeCP2 T308A knock-in mouse model and the analysis of these mutant mice indicated that it contributes to some of the neurological deficits in RTT [85]. Yet, it is still unclear whether the mutation of residue R306 has an influence on chromatin structure.

In addition to missense mutations, several nonsense RTT mutations have been described within the ID or the TRD. In general, these truncations showed decreased protein stability in vivo and DNA binding affinity in vitro [141].

MeCP2 R168X generates a truncated protein with a deletion of the complete TRD and C-terminal region. Male and female mice with R168X expression showed typical RTT phenotype, but little is known about the underlying mechanism. Although the entire MBD is retained, MeCP2 R168X has impaired ability to form higher order structures as tested by in vitro nucleosomal array (NA) assays [140].

The functional importance of the MeCP2 AT-hooks is highlighted by a comparative study in mice expressing either MeCP2 R270X or MeCP2 G273X (a truncation found in only one male RTT patient), which yielded a different developmental rate and phenotypic progression [148]. MeCP2 R270X mutant mice survived less time than MeCP2 G273X (85 days and 201 days, respectively) due to a disrupted AT-hook 2 (aa 264-273) in the MeCP2 R270 truncation. AT-hook 2 disruption decreased the ability of MeCP2 to promote oligomerization of NA in vitro and mislocalization of chromatin-remodeling protein ATRX in vivo [144].

In summary, the severe phenotypes of RTT patient mutations described above emphasize how essential protein stability, DNA/methyl cytosine binding, interactions with other proteins and ultimately chromatin organization are for proper MeCP2 function in vivo.

## 3. MeCP2 in Higher Order Chromatin Compartmentalization

MeCP2 is a multifunctional epigenetic reader regulated at multiple levels including, as reviewed above, specific isoforms, interacting factors, post-translational modifications and their interplay within the chromatin context. Yet, it is not well understood how MeCP2 orchestrates genome architecture. In this section, we will summarize findings related to the role of MeCP2 on higher order chromatin organization and propose a unifying model.

### 3.1. MeCP2 and Chromatin Looping

MeCP2 was described to compact nucleosomal arrays (NAs) [140] and to form loops involving undersaturated (DNA partially occupied by nucleosomes) nucleosomal arrays in vitro [24]. While wildtype MeCP2 was shown to form nucleosome-MeCP2-nucleosome ‘sandwich’ structures bringing two nucleosomes closely together, the RTT truncation mutant R294X was shown to form DNA-MeCP2-DNA ‘stem’ motifs, bringing nucleosome entry and exit site in close proximity [24]. Interestingly, the RTT mutation R106W, which does not bind to methylated DNA (see Table 3), did not induce any chromatin conformations. Thus, MeCP2 loop formation was proposed to proceed in a two step process involving methylation-dependent DNA binding followed by methylation-independent interactions between MeCP2 CTD and nucleosomes [24]. Of note, MeCP2 was also shown to bind to four-way junction DNA, which has a similar conformation as the ‘stem’ motif [24,149]. Importantly, MeCP2 was proposed to be involved in the formation of a silent chromatin loop at the imprinted *Dlx5-Dlx6* locus, and this loop is lost in RTT [150].

Current models though propose that the chromatin loops are promoted by ‘loop extrusion’, where cohesin extrudes chromatin until it encounters boundaries created by CTCF (CCCTC-binding factor) binding [151,152], albeit the underlying mechanism is unclear. MeCP2 has been reported to interact with ATRX and cohesin subunits SMC1 (structural maintenance of chromosomes protein 1) and SMC3 using coimmunoprecipitation experiments in mouse forebrain [64]. ATRX was proposed to create an extended DNA linker region for CTCF binding [153], and CTCF was reported to promote loop formation together with the cohesin complex [154,155]. Of note, the interaction of MeCP2 with cohesin subunit SMC3 was found to be induced by S229 phosphorylation and inhibited by the S80 phosphorylation of MeCP2 [19], indicating a role of MeCP2 and its modifications on chromatin looping.

Contrary to MeCP2, it was frequently described that CTCF shows a decreased binding affinity to methylated DNA [156,157]. Wang et al. found based on DNA methylome data from 13 cell types that immortalized cells displaying DNA hypermethylation had elevated CTCF level [158]. This might constitute a compensatory mechanism for lower CTCF binding due to hypermethylation [158] and may rescue CTCF mediated insulation of known tumor suppressor genes against methylation dependent silencing [159,160]. Furthermore, the 5mC oxidation product 5caC was found to enhance CTCF association to DNA and facilitate binding to low affinity CTCF binding motifs [161,162]. As 5mC oxidation to 5hmC followed by further oxidation to 5fC and 5caC was proposed to enable cytosine demethylation ([163], see above), CTCF association to 5caC hints to a CTCF-based mechanism reinforcing its own binding [162]. As MeCP2 has been shown to protect 5mC from TET mediated oxidation [44], MeCP2 might, thus, influence CTCF binding and DNA loop formation.

As a conclusion, the potential structural and functional interactions between MeCP2 and CTCF are still poorly understood and need to be clarified in further studies, especially considering the importance of both proteins in regulation of chromatin structure and gene expression. Mechanistically, loop formation has been proposed to give rise to TADs, whose boundaries are at least in part defined by CTCF and cohesin [164]. Although there is no evidence directly showing any effects of MeCP2 on TADs, it is still noteworthy to explore if and how MeCP2 organizes TADs, considering the role of MeCP2 on chromatin looping and counteracting CTCF binding.

### 3.2. MeCP2 and Heterochromatin Compartmentalization

Quantification of MeCP2 in neurons showed it to be nearly as abundant as histone octamers [31]. In MeCP2 deficient neurons, the level of histone H1 doubled [31], whereas in wild type neurons, the H1 level was half of the amount of H1 in other cells [165], indicating that MeCP2 acts as a histone H1-like chromatin linker. Accordingly, MeCP2 was shown: to accelerate H1 exchange in vivo, hence decreasing dwell time of histone H1 in chromatin [166]; to have a similar mobility to H1 in vivo; and to share with H1 an overlapping binding site on nucleosomes in vitro [31,166,167,168]. In fact, by in vitro fluorescence anisotropy assays, it was observed that MeCP2 could replace histone H1 from chromatin [166,169] and globally alter the chromatin state. MeCP2 deficiency was also reported to affect global chromatin composition and state by increasing H3 acetylation [31]. Hence, MeCP2 was proposed to dampen transcriptional noise from repetitive DNA elements including satellite DNA in a DNA methylation-dependent manner [31]. MeCP2 was also shown to increase H3K9me2 at the promoter of the *SIRT1* gene [170] and MeCP2 inhibition was shown to decrease H3K27me3 levels on silenced gene promoters [171], indicating a role of MeCP2 in facultative heterochromatin regulation. On the other hand, MeCP2 was also reported to activate gene expression by binding the transcription activator CREB1 in euchromatin as mentioned above [17].

Based on the cytological analysis of DNA condensation level, eukaryotic chromatin can be broadly divided into the actively transcribed, open euchromatin and the densely packed, repressed heterochromatin. Heterochromatin is rich in methylated cytosines, which can be specifically recognized by multiple epigenetic readers including MeCP2.

In vivo MeCP2 was shown to be enriched at pericentric heterochromatin [4]. Pericentric heterochromatin is localized in proximity to the centromere and enriched in AT-rich major satellite DNA repeats occupying about 10% of the mouse genome [172]. In the interphase nucleus, different pericentric heterochromatin regions were shown to fuse and form locally extremely condensed regions called chromocenters [173], a distinct, supra-chromosomal, membraneless heterochromatin domain also enriched in HP1 and H3K9me3. As MeCP2 was shown to interact with HP1 and to colocalize with HP1 in heterochromatin [51] (Table 1), this enables a cross talk between histone methylation and DNA methylation pathways strengthening heterochromatin formation. The influence of MeCP2 in chromocenter organization was demonstrated by Brero et al. [174], showing that, during myogenic differentiation, the number of chromocenters decreased, i.e., heterochromatin clustered into larger compartments, concomitantly with increased MeCP2 level and genome methylation. Of note, ectopic MeCP2-YFP could promote pericentric heterochromatin clustering even in the absence of cellular differentiation.

Expanding from this initial study, the role of MeCP2 during neuronal differentiation was analyzed comparing wild type and MeCP2 deficient mouse embryonic stem cells [175]. An increased MeCP2 level and enrichment at chromocenters was measured during neuronal differentiation, together with significant chromocenter clustering. Accordingly, the chromocenter clustering function was impaired in the MeCP2 deficient mouse embryonic stem cells. Furthermore, ectopic expression of MeCP2 with RTT mutations showed impaired heterochromatin accumulation and decreased chromatin clustering function [28], suggesting a role of heterochromatin organization in RTT.

At the molecular level, using in vitro nucleosomal arrays, Georgel et al. in 2003 observed by electron microscopy that NAs formed both extensively condensed ellipsoidal particles and oligomeric suprastructures upon addition of MeCP2. This was independent of DNA methylation and relying upon regions downstream of MBD, as R168X truncation mutant failed to assemble oligomeric suprastructures [140,176]. This was further confirmed by the observation that the ID, TRD and CTD alpha could bind and compact NAs and that R270X and R273X, truncated within the TRD and missing the whole CTD, could not compact and oligomerize NAs [23,144]. These facts could in part explain how nonsense mutations of MeCP2 lead to severe symptoms of RTT.

It is still far from clear how MeCP2 organizes heterochromatin structure, but emerging evidence suggests a role of phase separation in heterochromatin condensation.

### 3.3. Phase Separation and Heterochromatin Condensation

Compartmentalization of heterochromatin within the cell nucleus is evolutionarily conserved. Recent evidence indicates that in eukaryotic cells, non-membrane bound compartments are present in both the cytoplasm (e.g., stress granules [177]) and the nucleus (e.g., nucleoli [178]) and chromocenters [174]. Although described decades ago, how such membraneless compartments dynamically form and function has been unclear. In 2009, Brangwynne et al. [179] proposed that germline P granules are liquid droplets with fast exchange dynamics, fusion and fission properties and round appearance formed by liquid–liquid phase separation [180], suggesting a possible mechanism for chromatin organization.

Proteins that could undergo phase separation often contain intrinsically disordered regions (IDRs) or low complexity regions (LCRs) [181]. Chemically, the process is based on weak forces (mostly hydrophobic interactions) and multiple electrostatic interactions including charge-charge, charge–π, π–π stacking interactions and hydrogen bonds [182,183,184]. Recent work implicates liquid-liquid phase separation in the nuclear organization, leading to the formation of various subdomains with distinct properties.

An earlier in vitro cryo-electron microscopy study of purified simian virus 40 minichromosome showed that the purified viral minichromosome was condensed into 10 nm globules. In high-salt buffer, these globules showed the ability to fuse, whereas at low salt conditions, they opened into filaments and nucleosome strings [185]. Maeshima et al. observed that NAs self-associate into globular oligomers in a cation-induced manner, which can be modulated by histone H1 and linker DNA [186]. Altogether, these studies suggest a ‘liquid drop’ model of chromosome organization.

More recently, NAs were shown to undergo histone tail dependent liquid-like phase separation in physiologic salt conditions, a phenomenon promoted by histone H1, controlled by linker DNA length and disrupted by histone acetylation [187]. Furthermore, NAs with acetylated histones could form a new liquid phase with multi-bromodomain proteins, and these droplets had distinct properties compared to droplets formed by unmodified histones.

Two recent studies found that HP1alpha protein could drive chromocenter formation via phase separation [188,189], linking phase separation to chromocenter structure and dynamics via multivalent interactions. Interestingly, MeCP2 was shown to have a highly unstructured nature [33] and to induce the formation of very large heterochromatin clusters when compared with HP1 [174]. Altogether, these studies suggest a framework to understand chromatin compartmentalization based on liquid–liquid phase separation.

### 3.4. Model for MeCP2 Function in Chromocenter Clustering

**Figure 3:**
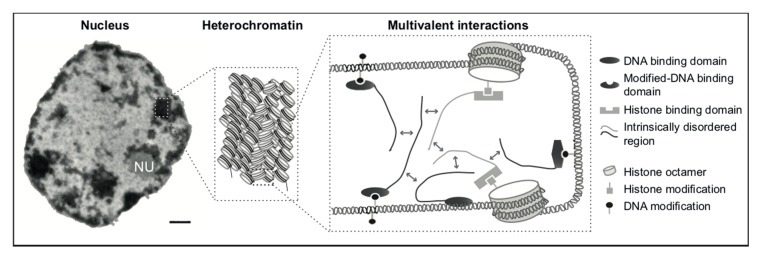
Role of multivalent interactions in heterochromatin formation. Left: transmission electron microscopy image of a mouse liver cell nucleus at interphase with electron dense regions corresponding to heterochromatin and electron light regions corresponding to euchromatin. NU: nucleoli. Scale bar = 0.5 µm. Middle: graphical representation of heterochromatin. Nucleosomes are tightly packed within heterochromatin and show limited accessibility to multiple factors binding DNA. Right: cartoon showing multivalent interactions that promote heterochromatin formation. Chromatin compaction can be maintained by multivalent interactions involving DNA and multiple proteins. Unmethylated, methylated DNA and posttranslational modifications of histones could recruit multiple protein factors containing intrinsically disordered regions (IDR) (like MeCP2 and HP1). Via these IDR regions multivalent homo and hetero weak interactions take place, promoting the formation of heterochromatin possibly by phase separation.

In summary, as described in the sections above, MeCP2 interacts with DNA, methyl cytosines and nucleosomes via separate domains, and interacts with several chromatin proteins. Furthermore, MeCP2 can replace linker histone H1 and has a highly unstructured nature. Firstly, like most proteins that could form liquid phase separation, MeCP2 intrinsically disordered regions consist of mainly positively charged residues (arginines, histidines and lysines). These residues form electrostatic interactions with the negatively charged amino acids in other proteins and phosphates in DNA or RNA, thus, building multivalent protein-protein/DNA/RNA interactions. Such interactions locally enrich or deplete factors in a dynamic manner, while being sensitive to post-translational modifications (as described above). Secondly, MeCP2 foci exhibit liquid-like properties in vivo. Brero et al. showed that MeCP2 forms round-shaped foci within the cell nucleus and foci in close proximity tend to fuse over time. Furthermore, during mitosis, these chromatin clusters undergo fission and reform again after cells have divided. MeCP2 was also shown to promote chromocenter clustering in a dose dependent manner [174]. In addition, purified MeCP2 protein alone showed oblate ellipsoid appearance in electron microscopy analysis [24]. Hence, and as depicted graphically in Figure 3, we propose that the multivalent interactions with proteins and DNA/nucleosomes, together with its ability to oligomerize and possibly create by itself phase separated compartments, altogether contribute to the in vivo ability of MeCP2 to dynamically and efficiently cluster and compartmentalize heterochromatin.

## Figures and Tables

**Table 1:** MeCP2 interaction partners and function upon interaction.

	Interactor	MeCP2 Function Upon Interaction	References
Transcriptional repression	HP.1	repression, formation of subcellular silencing compartments	Agarwal et al., 2007 [51]
	PU.1	formation of repression complex, possibly recruitment of mSin3A-HDAC	Suzuki et al., 2003 [15]
	Dnmt1	association with MeCP2 contributes to maintenance methylation	Kimura & Shiota 2003 [53]
	LANA	MeCP2 directs LANA to chromocenters, might contribute to LANA-mediated repression	Matsumura et al., 2010, Krithivas et al., 2002 [62,63]
	ATRX	targeting to heterochromatic regions in mature neurons, silencing of imprinted genes; possibly control of nucleosome positioning	Nan et al., 2007, Kernohan et al., 2010 [57,64]
	Sin3A	transcriptional repression, corepression complex with HDAC and MeCP2	Nan et al., 1998, Jones et al., 1998 [11,12]
	YY1	cooperation in repression	Forlani et al., 2010 [16]
	c-Ski	transcriptional repression	Kokura et al., 2001 [14]
	MBD2	heterointeractions, might increase heterochromatin clustering	Becker et al., 2013 [56]
	MeCP2	homointeractions, might increase heterochromatin clustering	Becker et al., 2013 [56]
	N-CoR	recruitment of N-CoR/SMRT to methylated DNA, bridge function of MeCP2	Kokura et al., 2001, Lyst et al., 2013 [14,20]
	Brahma	transcriptional repression	Harikrishnan et al., 2005 [65]
	CoREST	transcriptional repression possibly involving REST, CoREST, MeCP2, SUV39H1 and HP1	Lunyak et al., 2002 [13]
	CREB	transcriptional activation	Chahrour et al., 2008 [17]
	LEDGF/p75	might differentially influence gene activation	Leoh et al., 2012 [18]
	SMC1, SMC3	interaction with MeCP2, ATRX, might promote repression by loop formation	Kernohan et al., 2010, Gonzales et al., 2012 [19,64]
RNA interaction	Prpf3	RNA binding, possibly involved in splicing	Long et al., 2011 [66]
	mRNA, siRNA	not known	Jeffrey et al., 2004 [67]
	YB-1	RNA-dependent complex, regulation of splicing	Young et al., 2005 [60]
	Sdccag1	not known	Long et al., 2011 [66]
	FBP11	not known	Buschdorf & Stratling 2004, Bedford et al., 1997 [58,59]
	HYPC	not known	Buschdorf & Stratling 2004 [59]
post-translational modifiers	H3K9 MT	targeting of histone methylation to methylated DNA	Fuks et al., 2003, Lunyak et al., 2002 [13,48]
	SUV39H1	association with MeCP2 might contribute to silencing by methylation of H3K9, creating HP1 binding sites	Lunyak et al., 2002 [13]
	HDAC 1/2	histone deacetylases form corepression complex with MeCP2 and Sin3A	Nan et al., 1998, Jones et al., 1998 [11,12]
	HIPK2, HIPK1	kinases might phosphorylate MeCP2 on S80 and S216	Bracaglia et al., 2009, Lombardi et al., 2017 [68,69]
	PARP	poly(ADP-ribosyl)ation reduces MeCP2 heterochromatin clustering ability	Becker et al., 2016 [70]
	CDKL5	association in vitro, phosphorylation of MeCP2 by CDKL5 unclear (opposing results in the two publications)	Mari et al., 2005, Lin et al., 2005 [71,72]

**Table 2:** Summary of MeCP2 post-translational modifications.

	Residue*	Modification	Species	MS/Other Methods	References**
**NTD**	K12	ubi	human	x/-	Gonzales et al., 2012 [19]
	S13	phos	human, mouse	x/-	Gonzales et al., 2012, Humphrey et al., 2013, Shiromizu et al., 2013 [19,74,75]
	S53	phos	human	x/-	Shiromizu et al., 2013, Bian et al., 2014, Sharma et al., 2014 [75,76,77]
	S68	phos	mouse	x/-	Huttlin et al., 2010 [78]
	S70	phos	mouse, human	x/-	Huttlin et al., 2010, Mertins et al., 2016 [78,79]
	S78	phos	human, mouse, rat	x/-	Dephoure et al., 2008, Zanivan et al., 2008, Tweedie-Cullen et al., 2009 [80,81,82]
	S80	phos	human, mouse, rat	x/x	Zhou et al., 2006, Tao et al., 2009, Bracaglia et al., 2009 [68,83,84]
	K82	ubi	human	x/-	Gonzales et al., 2012 [19]
	S86	phos	mouse, human	x/x	Ebert et al., 2013, Mertins et al., 2014 [85,86]
**MBD**	R115	met	human	x/-	Geoghegan et al., 2015 [87]
	S116	phos	human	x/-	Dephoure et al., 2008, Kettenbach et al., 2011, Sharma et al., 2014 [77,80,88]
	K119	ubi, dimet	human	x/-	Gonzales et al., 2012, Jung et al., 2008 [19,89]
	Y120	phos	human, mouse	x/x	Dephoure et al., 2008, Bergo et al., 2015, D’Annessa et al., 2018 [80,90,91]
	K130	ubi	human	x/-	Wagner et al., 2011, Gonzales et al., 2012 [19,92]
	K135	ubi	human	x/-	Gonzales et al., 2012 [19]
	T148	phos	mouse	x/-	Tao et al., 2009 [84]
	S149	phos	mouse, human	x/-	Tao et al., 2009, Olsen et al., 2010, Kettenbach et al., 2011 [84,88,93]
	T160	phos	mouse	x/-	Tweedie-Cullen et al., 2009 [82]
	R162	met	mouse, human	x/-	Guo et al., 2014, Larsen et al., 2016 [94,95]
**ID**	163–206	PAR	human, mouse, rat	x/x	Jungmichel et al., 2013, Becker et al., 2016 [70,96]
	S164	phos	mouse	x/x	Tao et al., 2009, Tweedie-Cullen et al., 2009, Stefanelli et al., 2016 [82,84,97]
	S166	phos	mouse, human	x/-	Huttlin et al., 2010, Yi et al., 2014, Mertins et al., 2014 [78,86,98]
	S178	phos	human	x/-	Shiromizu et al., 2013 [75]
	T184	phos	human, mouse	x/-	Mertins et al., 2014 [86]
	T203	phos	human	x/-	Carrier et al., 2016 [99]
	S204	phos	human	x/-	Carrier et al., 2016 [99]
	K210	dimet	human	x/-	Jung et al., 2008 [89]
	S216	phos	human (mouse, rat)	x/x	Olsen et al., 2010, Kettenbach et al., 2011, Lombardi et al., 2017 [69,88,93]
	K219	acet	rat	x/-	Lundby et al., 2012 [100]
	K223	ubi	human	x/-	Akimov et al., 2018 [101]
	K223	SUMO	mouse	-/x	Cheng et al., 2014 [102]
	T228***	phos	human	x/-	Mertins et al., 2014 [86]
	S229	phos	human, rat (mouse)	x/x	Zhou et al., 2006, Chen et al., 2009, Gonzales et al., 2012 [19,83,103]
	K233	ubi	human	x/-	Gonzales et al., 2012 [19]
	244–275	PAR	human, mouse, rat	x/x	Jungmichel et al., 2013, Becker et al., 2016 [70,96]
	K249	ubi	human	x/-	Gonzales et al., 2012 [19]
	K256	ubi	human	x/-	Gonzales et al., 2012 [19]
	K267	met	human	x/-	Wu et al., 2015 [104]
**NID**	K271	ubi	human	x/-	Gonzales et al., 2012 [19]
	S274	phos	mouse (human)	x/x	Tweedie-Cullen et al., 2009, Humphrey et al., 2013, Ebert et al., 2013 [74,82,85]
	S292	phos	mouse, rat	x/x	Humphrey et al., 2013, Liu et al., 2015 [74,105]
	S295	phos	mouse	x/-	Humphrey et al., 2013 [74]
	K305	ubi	human	x/-	Gonzales et al., 2012 [19]
	K307	ubi, acet	human	x/-	Gonzales et al., 2012 [19]
	T308	phos	mouse	-/x	Ebert et al., 2013 [85]
**CTD**	T311	phos	mouse, human	x/-	Huttlin et al., 2010, Mertins et al., 2014, Parker et al., 2015 [78,86,106]
	S313	phos	human, mouse	x/-	Bian et al., 2014, Sharma et al., 2014, Parker et al., 2015 [76,77,106]
	K321	acet, ubi	human, mouse	x/-	Gonzales et al., 2012, Beli et al., 2012, Weinert et al., 2013 [19,107,108]
	T327	phos	human	x/-	Shiromizu et al., 2013 [75]
	S341	phos	mouse	x/-	Humphrey et al., 2013 [74]
	K347	met	human	x/x	Dhayalan et al., 2011, Wu et al., 2015 [109,110]
	S357	phos	human	x/-	Yang et al., 2006 [111]
	S359	phos	human	x/-	Yang et al., 2006, Bian et al., 2014 [76,111]
	S360	phos	human, mouse	x/-	Yang et al., 2006, Grimsrud et al., 2012, Humphrey et al., 2013 [74,111,112]
	S393	phos	human	x/-	Bian et al., 2014 [76]
	S399	phos	mouse, rat, human	x/-	Tao et al., 2009, Gonzales et al., 2012 [19,84]
	S421	phos	mouse, rat (human)	x/x	Zhou et al., 2006, Tao et al., 2009, Deng et al., 2010 [83,84,113]
	S424	phos	human, rat, mouse	x/x	Dephoure et al., 2008, Tao et al., 2009, Li et al., 2011 [80,84,114]
	T434	gl	rat, mouse	x/-	Wang et al., 2010, Alfaro et al., 2012, Trinidad et al., 2012 [115,116,117]
	T441	gl	mouse	x/-	Alfaro et al., 2012 [116]
	T443/T444***	gl	rat	x/-	Wang et al., 2010 [115]
	K447	acet	human	x/-	Choudhary et al., 2009, Beli et al., 2012, Wu et al., 2015 [104,107,118]
	T477	phos	human	x/-	Sharma et al., 2014 [77]
	S484	phos	human, mouse	x/-	Kettenbach et al., 2011, Schweppe et al., 2013, Mertins et al., 2014 [86,88,119]

Modifications identified by mass spectrometry (MS) might have unclear localization. x means the method as listed above was used, - means it was not used. * modification numbering according to mouse MeCP2 isoform starting in exon 2 (mouse: 484 aa, human: 486 aa, rat: 492 aa) ** references only exemplary (for more information see PhosphositePlus.org) *** residue numbering according to species mentioned as it differs from mouse.

**Table 3:** Summary of high frequency RTT-related MeCP2 point mutations and phenotypes.

	Mutation	Frequency	Effect on: Mice, Cell, Protein	References
**MBD**	R106W	132	Protein: Abolished methyl-DNA binding ability.	Ballestar et al., 2000 [126]
	R106Q	21	Protein: Reduced methyl-DNA binding ability.	Yang et al., 2016 [127]
	R133C	217	Mice: Decreased life span of 42 weeks and body weight. Protein: Reduced chromatin binding ability.	Brown et al., 2015 [128]
	S134C	21	Protein: Decreased stability and folding, reduced methyl-DNA binding.	Yang et al., 2016 [127]
	A140V	28	Mice: Late onset cognitive regression, pyramidal symptoms, parkinsonism, and bipolar symptoms. Increased cell packing density, abnormal dendritic branching of neurons. Life span: >14 months. Cell: Smaller neuron size. Down-regulation of the mTOR signaling pathway. Protein: Increased folding stability.	Venkateswaran et al., 2014 Jentarra et al., 2010 Ma et al., 2014 Sampathkumar et al., 2016 Yang et al., 2016 [127,129,130,131,132]
	P152R	71	Protein: Decreased stability and folding, reduced methyl-DNA binding.	Yang et al., 2016 [127]
	T158M	419	Mice: Decreased life span of 13 weeks and body weight. Disturbed nucleolin subcellular localization. Cell: Reduced neurite outgrowth, reduced dendritic complexity, and impaired mitochondrial health in forebrain neurons, reduced CREB and phosphorylated CREB levels. Protein: Decreased protein stability and methyl-DNA binding ability.	Lundvall et al., 2006 Olson et al., 2018 Bu et al., 2017 Chapleau et al., 2009 Brown et al., 2015 [128,133,134,135,136]
**ID**	R168X	364	Mice: Breathing dysfunction, hind limb clasping and atrophy, hypoactivity. Decreased life span of ~12 weeks. Male mice: Impaired motor and cognitive function and reduced anxiety, abnormal hypoxic and hypercapnic responses, apnea incidence, irregular breath cycle and decreased breathing rate, enriched outside chromocenters. Protein: Decreased chromatin compaction ability, decreased methyl-DNA binding.	Lawson-Yuen et al., 2007 Schaevitz et al., 2013 Bissonnette et al., 2014 Georgel et al., 2003 Yusufzai et al., 2000 [137,138,139,140,141]
	R255X	313	Mice: Decreased brain weight, increased breathing, incidence of arrhythmia, anxiety, motor and learning impairments. Cell: mTORC1 pathway abnormalities, decreased nucleolin level, increased phosphorylation of mTORC2 (S2481) and mTORC1 (S2448). Protein: Decreased methyl-DNA binding.	Pitcher et al., 2015 Olson et al., 2018 Yusufzai et al., 2000 [134,141,142]
**NID**	R270X	274	Male: Severe neonatal encephalopathy and death before 4 years of age. Mice: Median life span of 85 days, increased body weight, decreased brain weight. Cell: Less athalassemia/mental retardation syndrome X linked (ATRX) foci. Protein: Decreased methyl-DNA binding, failed to form a higher order structure with nucleic acids and reduced activity to oligomerize nucleic acids.	Villard et al., 2007 Baker et al., 2013 Yusufzai et al., 2000 [141,143,144]
	R294X	237	Cell: Induce caspase mediated apoptosis, rescued by FoxG1. Protein: Decreased methyl-DNA binding; decreased stability.	Lundvall et al., 2006 Yusufzai et al., 2000 [133,141]
	R306C	245	Mice: Hind limb clasping, impaired mobility and motor coordination, reduced brain weight and size. Cell: Loss of interaction with NCoR/SMRT. Protein: Loss of T308 phosphorylation.	Lyst et al., 2013 [20]

X means point mutation generating a truncated protein. Mutation numbering according to human MeCP2 isoform starting in exon 2.

## Abbreviations

5caC	5-carboxycytosine
5fC	5-formylcytosine
5hmC	5-hydroxymethylcytosine
5mC	5-methylcytosine
acet	acetylation
ATRX	a-thalassemia/mental retardation syndrome X linked
C	cytosine
CREB	cyclic AMP-responsive element-binding protein
CTCF	CCCTC-binding factor
CTD	C-terminal domain
(di)met	(di)methylation
gl	N-acetylglucosamine
HDAC	histone deacetylase
HP1	heterochromatin protein 1
ID	intervening domain
MBD	methyl-CpG-binding domain
MeCP2	methyl-CpG binding protein 2
NA	nucleosomal array
NID	NCoR/SMRT interaction domain
NTD	N-terminal domain
PAR	poly(ADP-ribosyl)ation
phos	phosphorylation
PTM	post-translational modification
RTT	Rett syndrome
TAD	topologically associated domain
TET	Ten-eleven translocation
TRD	transcriptional repression domain
ubi	ubiquitination
YB-1	Y box-binding protein 1
